# The Impact of Trust in Health Information Sources on Cancer Beliefs Among Older Adults

**DOI:** 10.1093/geroni/igaf122.3210

**Published:** 2025-12-31

**Authors:** Jonathan Nutakor

**Affiliations:** School of Public Administration, Guizhou University of Finance and Economics, Guiyang, Guizhou, China (People’s Republic)

## Abstract

Beliefs in cancer prevention and causation shape older adults’ health behaviors. Trust in sources of health information, i.e., doctors, family, and government, may affect these beliefs. This study explores the association between trust in health information sources and cancer beliefs in older adults. Data was analyzed from older adults (60+) in the Health Information National Trends Survey (HINTS) 5. Logistic regression models were used to test for relationships between trust levels and cancer beliefs, adjusting for demographic factors. Respondents aged 85+ were more likely to believe that everything causes cancer (Coef.=0.775, p = 0.034). Women were less likely than men to believe that everything causes cancer (Coef.=-0.306, p = 0.038). Those who were living as married were more likely to hold the belief that it is impossible to reduce cancer risk (Coef.=2.080, p = 0.044). Trust in doctors was inversely related to beliefs that anything causes cancer (Coef.=-0.242, p = 0.089). Belief in government was strongly related to reduced belief in the impossibility of risk reduction (Coef.=-0.436, p = 0.001). Trust in the government was negatively associated with willingness for behavioral change (Coef.=-0.588, p = 0.001), and trust in charities had a positive trend (Coef.=0.295, p = 0.072). In conclusion, trust in health information sources has a profound influence on older adult’s cancer-related beliefs and behavior. Lower trust in doctors and government sources is also associated with greater levels of cancer-related myths. Building trust in credible health sources could potentially augment cancer prevention in older adults.

